# Metabolomic Changes in the Rat Eye Lens During the Cataract Onset

**DOI:** 10.3390/molecules31122194

**Published:** 2026-06-22

**Authors:** Olga A. Snytnikova, Anton A. Smolentsev, Nataliya G. Kolosova, Anzhella Z. Fursova, Yuri P. Tsentalovich

**Affiliations:** 1 International Tomography Center, Siberian Branch of the Russian Academy of Sciences, Institutskaya Str. 3a, 630090 Novosibirsk, Russia; a.smolentsev@tomo.nsc.ru (A.A.S.); yura@tomo.nsc.ru (Y.P.T.); 2 The Federal Research Center Institute of Cytology and Genetics, Siberian Branch of the Russian Academy of Sciences, Academician Lavrentiev Avenue 10, 630090 Novosibirsk, Russia; kolosova@bionet.nsc.ru (N.G.K.); fursova@bionet.nsc.ru (A.Z.F.); 3 State Novosibirsk Regional Clinical Hospital, St. Nemirovich-Danchenko 130, 630087 Novosibirsk, Russia

**Keywords:** OXYS rats, lens, cataract, aging, metabolomic profile, NMR spectroscopy

## Abstract

This study aimed to characterize metabolomic changes in the eye lens of senescence-accelerated OXYS rats in comparison with control Wistar rats, and to identify biochemical shifts associated with genotype, age, and cataract progression. Cataract severity was clinically graded. Rats’ lenses were analyzed using quantitative ^1^H NMR spectroscopy at 3.6 and approximately 4.5 months of age. A total of 43 metabolites were quantified. We found that at 3.6 months of age, OXYS lenses exhibited a significant accumulation of 17 metabolites, primarily amino acids, compared to Wistar rats, suggesting an imbalance between amino acid uptake and crystallin biosynthesis. However, by 4.5 months, OXYS lenses exhibited rapid metabolic changes characterized by significant decreases in amino acid, glucose, and key energy/antioxidant markers, including NAD, adenylate energy charge, and hypotaurine. Clinical cataract grade (Grade 2 vs. 3) had a negligible impact on the overall metabolomic profile. Our results indicate that profound metabolic reorganization, including an initial amino acid excess followed by energy and antioxidant depletion, precedes the morphological manifestation of cataracts in OXYS rats. We suggest that a biochemical “point of no return” occurs early in cataractogenesis, while subsequent increase in lens opacification is a secondary consequence of preexisting metabolic disturbances.

## 1. Introduction

We acquire over 80% of environmental information through the visual system, which makes ocular health a primary determinant of the quality of life. Among the leading causes of visual impairment are both natural aging and the development of pathological processes, with cataracts (lens opacification that obstructs light transmission to the retina) being the most prevalent. Despite the high incidence of cataracts, the molecular mechanisms underlying cataractogenesis remain incompletely understood. Consequently, effective conservative prevention and therapy methods are currently lacking, and the only radical treatment remains surgical extraction of the affected lens with subsequent intraocular lens implantation.

The development of non-invasive therapies, early diagnostic tools, and preventive strategies are impossible without a detailed understanding of the biochemical processes occurring in lens tissues during aging and pathogenesis. In this context, metabolomics has emerged as a promising multidisciplinary field in ophthalmology, aimed at identifying diagnostic biomarkers, investigating specific metabolite groups, elucidating molecular mechanisms of pathogenesis, and identifying potential pharmacological targets [1,2,3,4,5,6,7,8,9].

However, the application of metabolomic profiling to the human lens is significantly limited by the scarcity of intact control samples from healthy tissues, which complicates the identification of specific differences between normal and pathological states. An alternative solution to this problem is the use of appropriate biological models. A relevant model of senile cataract is the senescence-accelerated OXYS rat strain [10,11]. This strain is characterized by an accelerated aging rate and the early onset of age-associated pathologies. While clinical signs of cataracts are absent in 20-day-old animals, the incidence reaches 100% by the age of 3 months. In 80% of cases, nuclear cataracts of varying degrees of maturity develop, ranging from initial opacities and nuclear compaction to the formation of a dense yellow nucleus. In terms of clinical manifestations and morphological features, cataractogenesis in OXYS rats corresponds to the progression of senile cataract in humans.

Previously, our laboratory conducted detailed quantitative metabolomic analyses of lenses from different animals and humans [12,13]. Evaluation of age-related changes in the lens metabolome of Wistar (control) and OXYS (cataract model) rats revealed that the concentrations of most metabolites decrease with age [12]. This decline is most pronounced between 1 and 3 months of age, which likely corresponds to the completion of lens maturation by the first month and the high growth rate of the young lens.

A comparative analysis of age-matched OXYS and Wistar rats [12] showed statistically significant differences observed for tryptophan, tyrosine, carnitine, glycerophosphate, reduced (GSH) and oxidized (GSSG) glutathione: their levels in OXYS lenses were, on average, 30% higher than in Wistar lenses. Conversely, choline concentrations were lower in the OXYS model. Elevated levels of tryptophan and GSH in OXYS rat lenses were also previously reported [10,14]. While the underlying causes of these effects remain unclear, for some metabolites, they may represent a compensatory response to oxidative stress.

In the present work, we performed a quantitative ^1^H nuclear magnetic resonance (NMR) analysis of rat lenses to identify changes occurring during the period of active cataract development. The specific objectives of the study were to compare the lens metabolome of OXYS rats at different stages of cataractogenesis, to identify metabolomic differences between intact and pathological lenses, and to determine general patterns of metabolomic changes during cataract development.

## 2. Results

### 2.1. Quantitative Metabolomic Profiling of Rat Lenses

A total of 43 water-soluble metabolites were identified and quantified in each lens sample, including amino acids, organic acids, antioxidants, osmolytes, glycosides, as well as purine and pyrimidine derivatives. The complete list of metabolites and their concentrations across the study groups is provided in Table S1. For each group, the mean concentrations and variation ranges were determined; these data are further visualized using box plots (Figure S1).

The measured metabolite concentrations spanned three orders of magnitude, ranging from 4.6 nmol/g to 7.5 μmol/g. The major components (with concentrations exceeding 1 μmol/g) in the rat lens were identified as formate, glucose, glutamate, glycerophosphocholine, reduced glutathione (GSH), hypotaurine, lactate, myo-inositol, phosphocholine, and taurine.

### 2.2. Multivariate Analysis of Metabolomic Profiles

**Figure 1:**
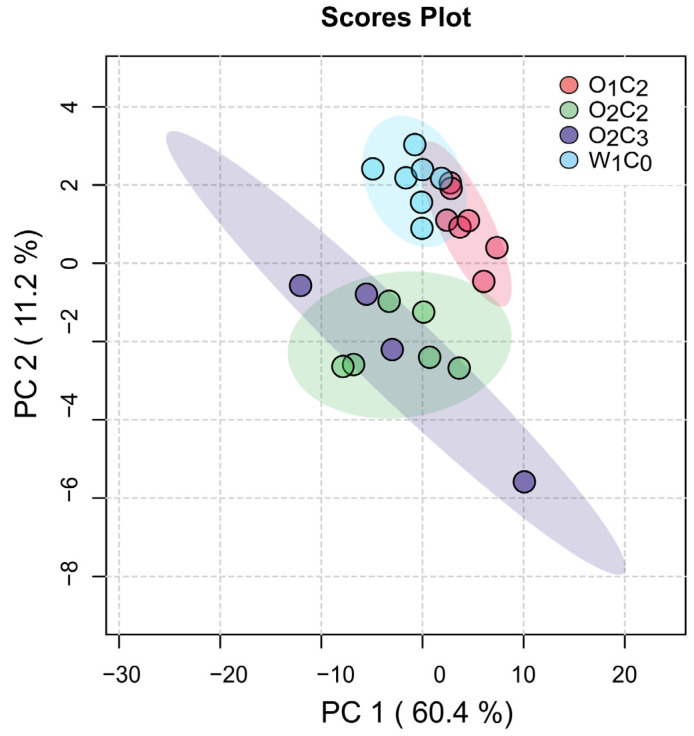
PCA scores plot of the concentration of water-soluble metabolites extracted from the lenses of Wistar and OXYS rats. Group designations: W (Wistar rats), O (OXYS rats); subscripts: 1 (3.6 months of age), 2 (approx. 4.5 months of age); C cataract; subscript: grade 0–3.

To assess the overall differences between the experimental groups, multivariate chemometric analysis was performed. Principal Component Analysis (PCA) was initially employed to visualize the distribution of all 43 metabolites across the samples (Figure 1). The PCA scores plot demonstrates that the second principal component (PC2, 11.2%) primarily accounts for the separation of samples based on the age of the animals. Groups aged 3.6 months (W_1_C_0_ and O_1_C_2_) form a distinct cluster from the older groups (approx. 4.5 months). Notably, no clear separation was observed between OXYS rat groups of the same age but with different cataract grades (O_2_C_2_ vs. O_2_C_3_), suggesting that age-related changes may dominate over stage-specific metabolic shifts at these time points.

### 2.3. Comparative Analysis of Metabolomic Profiles: Healthy Versus Cataractous Lenses

**Figure 2:**
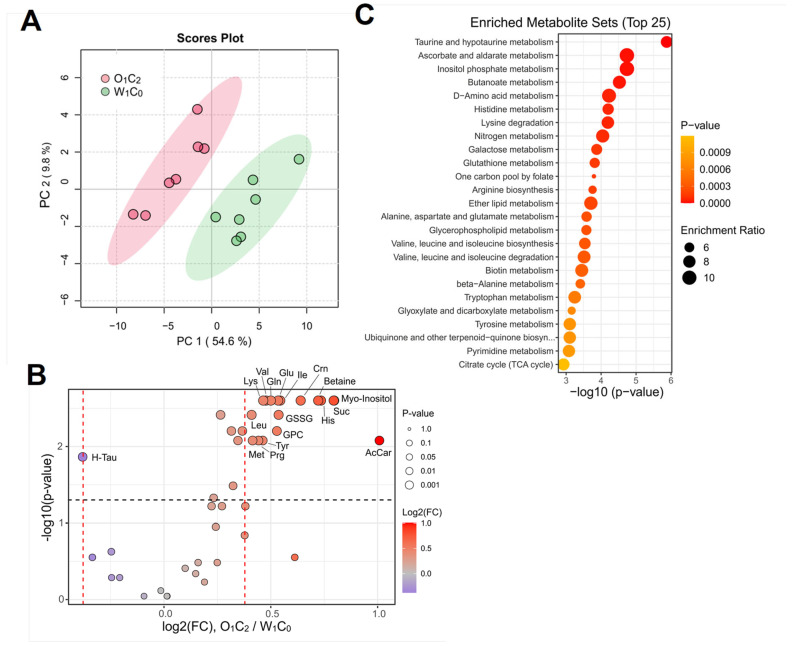
Comparative metabolomics of Wistar (control) and OXYS (Grade 2 cataract) rat lenses at 3.6 months of age: (**A**) PCA scores plot showing the separation of metabolomic profiles. Colored ovals indicate the 95% confidence regions; (**B**) Volcano plot comparing metabolite concentrations in O_1_C_2_ and W_1_C_0_ groups (*n* = 7 per group). Red dashed lines indicate an absolute fold change > 1.3; the black dashed line represents the *p* < 0.05 threshold with FDR adjustment. Differentially abundant metabolites are labeled; (**C**) Metabolite Set Enrichment Analysis identifying significant perturbed metabolic pathways in cataractous lenses. Abbreviations: AcCar—Acetylcarnitine; Crn—Creatinine; Glu—Glutamate; Gln—Glutamine; GPC—Glycerophosphocholine; GSSG—Oxidized glutathione; His—Histidine; H-Tau—Hypotaurine; Ile—Isoleucine; Leu—Leucine; Met—Methionine; Prg—Pyroglutamate; Suc—Succinate; Tyr—Tyrosine; Val—Valine; Lys—Lysine.

To identify metabolic signatures associated with cataract development, we compared the metabolomic profiles of 3.6-month-old Wistar rats (healthy controls) and age-matched OXYS rats (Grade 2 cataract). The divergence between these groups was visualized using Principal Component Analysis (Figure 2A) and Volcano plots (Figure 2B), which highlight statistically significant differences in metabolite concentrations.

As shown in the PCA scores plot (Figure 2A), the metabolic profiles of healthy and cataractous lenses exhibit distinct clustering, indicating substantial biochemical shifts. The Volcano plot analysis (Figure 2B) identified 18 differentially expressed metabolites (thresholds: *p* < 0.05, fold change > 1.3. In OXYS rat lenses, hypotaurine concentration was significantly depleted, whereas the levels of 17 other metabolites were significantly elevated. These upregulated compounds include: amino acids and their derivatives (glutamate, histidine, isoleucine, valine, lysine, betaine, glutamine, tyrosine, pyroglutamate, leucine, and methionine); energy metabolism intermediates (creatinine, acetylcarnitine, and succinate) and other key metabolites, such as glycerophosphocholine (GPC), oxidized glutathione (GSSG), and myo-inositol.

To elucidate the biochemical pathways perturbed during cataractogenesis, we performed Metabolite Set Enrichment Analysis (MSEA) comparing the 3.6-month-old Wistar and OXYS groups. The analysis revealed that 38 out of 43 analyzed pathways were significantly altered (*p* < 0.05). These perturbed pathways involved 35 of the 43 measured metabolites, with 24 metabolites showing statistically significant differences (*p* < 0.05). The top 25 significantly enriched metabolic pathways are presented in Figure 2C. The widespread nature of these changes suggests a systemic metabolic reorganization in the lens during the cataract progression.

### 2.4. Age-Related Metabolomic Shifts in OXYS Rats Lenses

**Figure 3:**
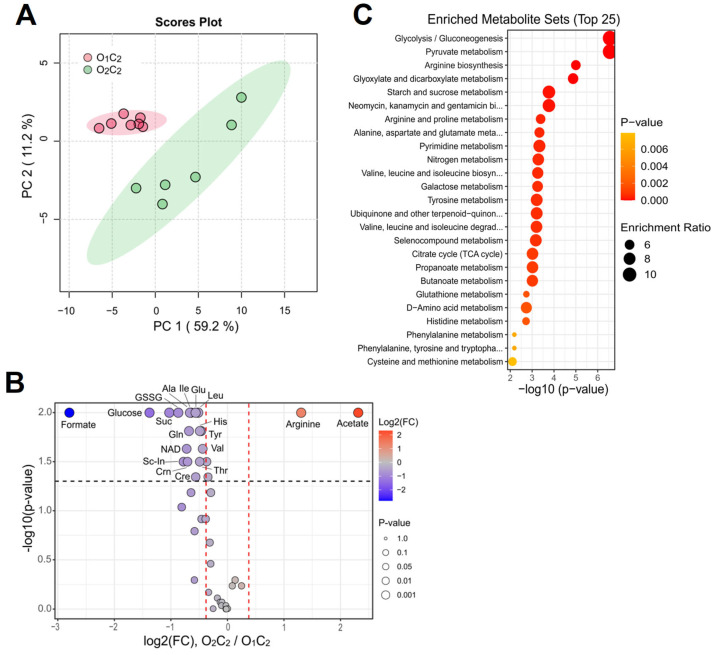
Comparative metabolomics of OXYS rat lenses (Grade 2 cataract) at 3.6 and 4.5 months of age: (**A**) PCA scores plot showing the separation of metabolomic profiles. Colored ovals indicate the 95% confidence regions; (**B**) Volcano plot comparing metabolite concentrations in O_2_C_2_ and O_1_C_2_ groups (*n* = 4–7 per group). Red dashed lines indicate an absolute fold change > 1.3; the black dashed line represents the *p* < 0.05 threshold with FDR adjustment. Significant differentially abundant metabolites are labeled; (**C**) Metabolite Set Enrichment Analysis identifying perturbed metabolic pathways. Abbreviations: Ala—Alanine; Cre—Creatine; Crn—Creatinine; Glu—Glutamate; Gln—Glutamine; GSSG—Glutathione oxidized; His—Histidine; Ile—Isoleucine; Leu—Leucine; NAD—Nicotinamide adenine dinucleotide; Sc-In—scyllo-Inositol; Suc—Succinate; Thr—Threonine; Tyr—Tyrosine; Val—Valine.

We further investigated the impact of aging on the lens metabolome by comparing 3.6-month-old and 4.5-month-old OXYS rats, both presenting with Grade 2 cataracts (Figure 3). Principal Component Analysis (PCA) revealed distinct age-related clustering (Figure 3A), indicating significant metabolic changes even within a short one-month interval. Volcano plot analysis (Figure 3B) identified 19 differentially abundant metabolites (thresholds: *p* < 0.05, fold change > 1.3). In the 4.5-month-old group, acetate and arginine concentrations were significantly elevated compared to the younger group. Conversely, the levels of 17 other metabolites were significantly depleted in the older animals. The majority of these differentially expressed compounds (Figure 3B) were amino acids, including alanine, glutamate, glutamine, histidine, isoleucine, leucine, threonine, tyrosine, and valine. Additionally, significant age-dependent decreases were observed for acetate, glucose, succinate, formate, creatine, creatinine, oxidized glutathione (GSSG), scyllo-inositol, and nicotinamide adenine dinucleotide (NAD).

Metabolite Set Enrichment Analysis (MSEA) demonstrated that 35 out of the 43 analyzed metabolic pathways were significantly altered (*p* < 0.05) between the two age groups. These perturbed pathways involved 29 of the 43 measured metabolites, with 20 metabolites showing statistically significant differences (*p* < 0.05). The top 25 significantly enriched metabolic pathways are shown in Figure 3C. These results suggest that aging in the OXYS model is associated with a widespread decline in the levels of key metabolites, despite the stable clinical grade of the cataract.

### 2.5. Impact of Cataract Grade on the Lens Metabolome

Further comparison of the metabolomic profiles within 4.5-month-old OXYS rats revealed no significant differences between animals of the same age but with different cataract severities (Grade 2 vs. Grade 3). The metabolomic compositions of the lenses at this age remained nearly identical regardless of the clinical stage (Figure S2). Univariate statistical analysis confirmed the absence of statistically significant differences in the concentrations of any measured metabolites. Furthermore, neither Volcano plot analysis nor Metabolite Set Enrichment Analysis (MSEA) identified any significant metabolic perturbations associated with the transition from Grade 2 to Grade 3 cataracts.

Detailed multivariate and univariate data for these comparisons are provided in the Supplementary Materials (Figures S1 and S2, Table S2).

### 2.6. Energy, Oxidation, and Glutamatergic Indices

**Figure 4:**
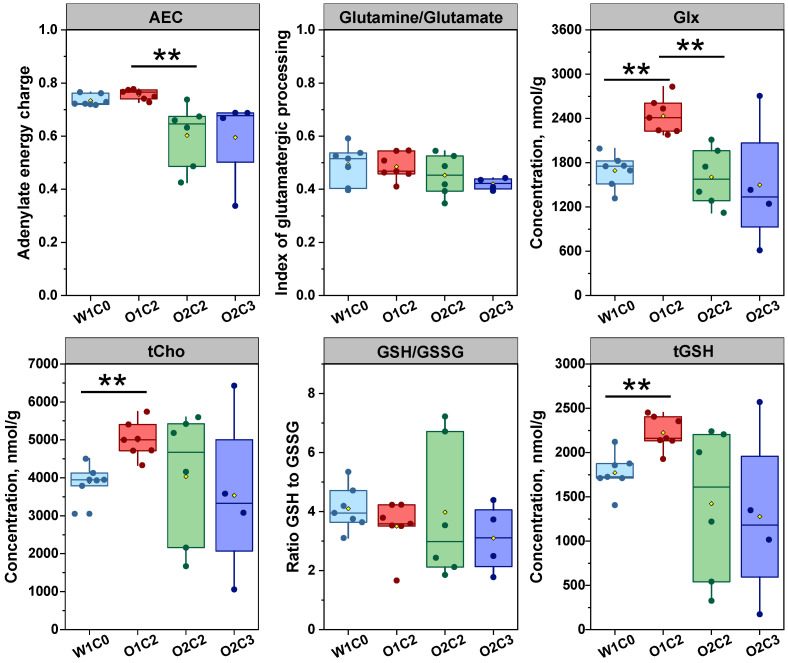
Metabolic indices during cataractogenesis and aging: AEC—Adenylate energy charge value; index of glutamatergic processing—ratio glutamine to glutamate; Glx—sum of glutamate and glutamine; tCho—sum of glycerophosphocholine, choline and phosphocholine; GSH/GSSG—glutathione/oxidized glutathione ratio; tGSH—sum of reduced and oxidized glutathione; GSSG—oxidized glutathione. The yellow point shows the mean value within the group. Box plots represent mean value and median ±1.5 IQR. The metabolite level in lenses is statistically different: **—*p* value < 0.01.

To better visualize the metabolomic differences between rat groups, we calculated the values of energy, oxidation, and glutamatergic indices, including adenylate energy charge (AEC), glutamine to glutamate ratio, GSH to GSSG ratio, total choline (tCho) and glutathione (tGSH), and the sum of glutamate and glutamine (Glx). The obtained results are collected in Table S4 and shown in Figure 4. Statistically significant differences were found for tCho, tGSH, and Glx when comparing the control group (intact lenses) and cataractous lenses in OXYS rats with Grade 2 cataracts. With age, a statistically significant difference was found for the AEC and Glx index.

## 3. Discussion

In the present study, we evaluated the metabolomic changes in rat lenses influenced by three primary factors: genotype (OXYS vs. Wistar), age (3.6 vs. 4.5 months), and cataract grade (Grades 2 vs. 3). Our findings indicate that genotype and age are the dominant factors shaping the lens metabolome, while the clinical grade of the cataract (at these specific stages) has a negligible impact. This suggests that the biochemical shifts associated with cataractogenesis in the OXYS strain occur near-simultaneously across the population, even if the morphological manifestation of opacities varies slightly in timing.

A notable finding is the significant accumulation of nearly all amino acids in 3.6-month-old OXYS lenses compared to age-matched Wistar controls (Figure 2). This shift affects the majority of metabolic pathways identified (Figure 2C). While elevated amino acid levels in OXYS lenses were previously noted [12], our data reinforce the hypothesis of a profound imbalance between amino acid uptake and utilization. The influx of amino acids from the aqueous humor, mediated by active transport in the lens epithelium [15,16], appears to exceed the rate of crystallin biosynthesis. A deceleration in protein synthesis during fiber cell differentiation in OXYS rats may lead to this “metabolic crowding,” potentially creating a proteotoxic environment that accelerates lens opacification.

Interestingly, between 3.6 and 4.5 months, the OXYS lens undergoes rapid metabolic changes. The levels of most amino acids (Ala, Glu, Gln, His, Ile, Leu, Tyr, Val) significantly drop, reaching values comparable to healthy Wistar rats. This rapid decline over just one month is remarkable. It likely reflects a deterioration of the lens transport system or a decrease in the transparency of the lens syncytium, hindering metabolite diffusion [17,18].

This “exhaustion” is further evidenced by the Adenylate Energy Charge (AEC) and NAD levels. A significant decrease in AEC and total glutathione (tGSH) in 4.5-month-old cataractous lenses compared to younger controls suggests an impending energetic crisis and a failure of antioxidant defense. The reduction in hypotaurine—a known antioxidant and osmolyte—further supports the role of oxidative stress as a driver of early-stage cataractogenesis.

Analysis of metabolic changes related to choline and glutamate abundances allowed for a deeper understanding of the pathophysiology of the disease. For example, we found a significant difference in total choline (tCho) between Wistar (W_1_C_0_) and OXYS (O_1_C_2_) lenses. Earlier, it was reported that aging and cataracts alternate the lipid composition of the human and animal lenses [19,20,21,22,23]. The observed difference in tCho level likely indicates the pathological changes in the lens cell membranes, leading to difficulty in transporting metabolites within the lens. For glutamatergic indices (Glx and Gln/Glu), we found, that the sum of glutamate and glutamine (Glx) was significantly lower in cataractous lenses. Since glutamate is a precursor for GSH, its depletion, combined with a decreased GSH/GSSG ratio, marks a critical failure in maintaining the thiol-redox balance [24,25,26]. Notably, only the Glx index showed a statistically significant age-related change (3.6 vs. 4.5 months) within the OXYS group, suggesting that glutamate-glutamine metabolism is highly sensitive to the progression of age-associated lens degradation and allowing Glx to be considered as an age marker.

The lack of significant differences between Grade 2 and Grade 3 cataracts suggests that the metabolic “point of no return” occurs prior to or at the onset of Grade 2. Once the systemic metabolic reorganization (characterized by NAD depletion and antioxidant failure) has taken place, further physical opacification may proceed as a downstream morphological consequence rather than a result of further acute metabolic shifts.

Several limitations of this study should be acknowledged. First, the metabolomic profiling was conducted within a relatively narrow age range (3.6 to 4.5 months); including earlier pre-cataractous stages and more advanced age groups would provide a more complete understanding of the metabolic trajectory of the disease. Second, the absence of age-matched Wistar controls for the 4.5-month-old group limits our ability to fully distinguish between strain-specific pathological changes and universal age-related metabolic decline. Third, while the whole-lens analysis provided a comprehensive overview, it may mask spatially-specific metabolic differences between the lens cortex and nucleus, where protein turnover and transport activities vary significantly. Finally, the relatively small sample sizes in certain subgroups may have limited the statistical power to detect subtle metabolic differences beyond the observed changes.

## 4. Materials and Methods

### 4.1. Animals

All animals were provided by the Center for Genetic Resources of Laboratory Animals at the Institute of Cytology and Genetics, Siberian Branch of the Russian Academy of Sciences (ICG SB RAS, Novosibirsk, Russia). The rats were maintained under standard laboratory conditions (12 h light/dark cycle, temperature 22 ± 2 °C, relative humidity 60%) with ad libitum access to water and a standard rodent diet (PK-120-1, Laboratorsnab, Moscow, Russia). Animal maintenance was supported by government-funded project (No. FWNR-2026-0025).

### 4.2. Ophthalmological Examination and Cataract Grading

The study involved male OXYS rats aged 3.6 and approximately 4.5 months, corresponding to the period of active clinical manifestation of cataracts. Age-matched male Wistar rats served as the control group. A comprehensive ophthalmological examination was performed by a specialist one day prior to lens collection. Mydriasis was induced using a 1% tropicamide solution.

Clinical evaluation was conducted using a Beta direct electric ophthalmoscope (Heine, Optotechnik, Gilching, Germany) with a manual slit-lamp attachment and a ShinNippon SL-45 slit lamp (Rexxam Co., Ltd., Tokyo, Japan). The severity of lens opacity was assessed according to the clinical classification and recommendations described in [27,28]. The pathological changes were graded as follows: Grade 0 corresponds to a clear lens (transparent); Grade 1—focal, delicate cortical or nuclear opacities (corresponding to 1–4 on the decimal scale); Grade 2—Manifest foci of opacity (corresponding to 5–8 on the decimal scale); Grade 3—intense opacity of the cortex or nucleus (corresponding to 9–10 on the decimal scale).

### 4.3. Experimental Groups

To investigate the dynamics of metabolomic profile changes during cataract progression, several animal groups were established (Table 1). The following nomenclature was adopted: O for OXYS rats and W for Wistar rats, C for cataract. The first subscript indicates the age group: 1 = (3.6 months) and 2 (approx. 4.5 months); the second subscript denotes the cataract grade; and *n* represents the total number of animals per group.

### 4.4. Sample Collection and Preparation

Animals were euthanized by CO_2_ inhalation in an unlined chamber, starting with room air and increasing the CO_2_ concentration at a rate sufficient to induce rapid anesthesia, with death occurring within 1 min. Immediately following decapitation, the eyeballs were enucleated, cleared of extraocular tissues, and incised along the limbus. The lenses were carefully extracted on ice and flash-frozen in liquid nitrogen. Each analytical sample consisted of both lenses from a single animal. Samples were stored at −70 °C until further analysis.

Extraction of water-soluble metabolites was performed using a methanol–chloroform–water protocol previously optimized in our laboratory [11]. Frozen lenses were weighed and homogenized in a cold (−20 °C) mixture of methanol, chloroform, and water (2:2:1 *v*/*v* ratio; 1200 μL of solvent per 80 mg of wet tissue). The homogenates were vortexed for 30 s, kept on ice for 10 min, and incubated at −20 °C for 30 min. To precipitate proteins, the samples were centrifuged at 12,000 rpm (4 °C) for 30 min. The upper hydrophilic fraction was collected and evaporated to dryness under vacuum. The dried extracts were reconstituted in 600 μL of 0.02 M deuterated phosphate buffer (pH 7.4) containing 20 μM sodium 2,2-dimethyl-2-silapentane-5-sulfonate (DSS) as an internal standard.

### 4.5. NMR Spectroscopy

^1^H NMR spectra were acquired using an AVANCE III HD 700 MHz spectrometer (Bruker BioSpin, Ettlingen, Germany) at the Center for Collective Use “Mass Spectrometric Investigations” SB RAS. All measurements were performed at 25 °C in 5 mm NMR tubes using a TXI ^1^H−^13^C/^15^N/D Z-gradient probehead. Spectra were recorded using a single-pulse zgpr sequence with the following parameters: a 70-degree detection pulse, a spectral width of 14 ppm, a relaxation delay of 5 s, and an acquisition time of 6.7 s. Water suppression was achieved through continuous wave presaturation during the relaxation delay. A total of 64 scans were accumulated for each sample. A typical ^1^H NMR spectrum of a rat lens is shown in Figure S3.

Data processing was performed using MestReNova v.12 (Mestrelab Research S.L., Santiago de Compostela, Spain), including manual phase and baseline correction. Chemical shifts were referenced to the DSS signal (0.00 ppm). Metabolite resonances were assigned by comparing experimental data with the Human Metabolome Database (https://hmdb.ca, accessed on 12 December 2025; Ref. [29]), spiking with reference compounds where necessary, and utilizing our previous metabolomic profiling data [11]. A complete list of identified metabolites, including their chemical shifts and multiplicities used for quantification, is provided in Table S2. Absolute concentrations were determined by integrating the peak areas relative to the internal standard (DSS), following the procedure described in [11].

### 4.6. Data Analysis and Statistics

Multivariate and univariate statistical analyses of the lens metabolomic profiles were performed on the MetaboAnalyst 6.0 web platform (https://metaboanalyst.ca, accessed on 12 February 2026; Ref. [30]) using normalized (autoscaled—mean-centered and divided by the standard deviation of each variable) concentration data. Principal Component Analysis (PCA), an unsupervised method, was applied to visualize data distribution and identify potential outliers.

For univariate analysis, intergroup comparisons were conducted using the non-parametric Wilcoxon rank-sum test. To control for multiple comparisons, *p*-values were adjusted using the Benjamini–Hochberg (FDR) procedure. Results were considered statistically significant at an adjusted *p* < 0.05. Metabolite Set Enrichment Analysis (MSEA) was further employed to identify impaired metabolic pathways. The MSEA plots were constructed with the Enrichment Analysis module of MetaboAnalyst 6.0 web-platform using the Kyoto Encyclopedia of Genes and Genomes database (KEGG; Ref. [31]). Detailed statistical outputs, including *p*-values and FDR-corrected values, are provided in the Supplementary Materials (Tables S1 and S3).

## 5. Conclusions

In this study, we investigated the changes in the lens metabolome during the progression of cataracts in OXYS rats. A comparative metabolomic analysis of Wistar and OXYS rat lenses across different ages and cataract grades revealed that genotype (OXYS vs. Wistar) and animal age are the primary factors determining the metabolic composition of the lens.

Notably, the clinical grade of the cataract (Grade 2 vs. Grade 3) for animals of the same strain and age had a negligible impact on the lens metabolome. These findings suggest that genetic predisposition and age-related metabolic shifts precede and dominate the biochemical landscape, while the morphological manifestation of cataracts (opacification) acts as a secondary consequence of these pre-established metabolic perturbations. Our data highlight that the major metabolic reorganization in cataractogenesis likely occurs at early stages, followed by a systemic decline in antioxidant and energetic resources, regardless of the subsequent rate of lens opacification.

## Figures and Tables

**Table 1:** Experimental design and characteristics of animal groups used for metabolomic profiling of rat lenses.

Group	W_1_C_0_	O_1_C_2_	O_2_C_2_	O_2_C_3_
*n*	7	7	6	4
Strain	Wistar	OXYS	OXYS	OXYS
Age (months)	3.6	3.6	4.6 ± 0.2	4.2 ± 0.3
Cataract Grade	0	2	2	3

## Data Availability

Raw NMR spectra, description of samples and metabolite concentrations are available at the Animal Metabolite Database repository, Experiment ID 303 (https://amdb.online/amdb/experiments/303/, accessed on 6 April 2026). All obtained data are available from the corresponding author upon request.

## Supplementary Materials

The following supporting information can be downloaded at: https://www.mdpi.com/article/10.3390/molecules31122194/s1, Figure S1: Concentrations of metabolites in the rat lenses; Figure S2: PCA scores plot demonstrating the distribution of metabolomic profiles between groups with different cataract severities (Grade 2 vs. Grade 3); Figure S3: Typical ^1^H NMR spectrum of a rat lens extract; Table S1: Concentrations of metabolites in the rat lens; Table S2: ^1^H NMR spectral data: chemical shifts and signal multiplicities of metabolites identified and quantified in lenses of Wistar and OXYS rats; Table S3: Wilcoxon rank-sum test on correlations of metabolite abundances in the experimental groups; Table S4: Metabolic indices for rat groups under study.
