# Supplementary material for: Metabolomic Changes in the Rat Eye Lens During the Cataract Onset
Source: Molecules. 2026 Jun 22;31(12):2194. doi: 10.3390/molecules31122194 (PMC13304499; doi:10.3390/molecules31122194)
Supplement: Supplementary file 1 [file molecules-31-02194-s001.zip › molecules-4347263-supplementary.pdf]

Metabolomic changes in the rat eye lens during the cataract onset

Olga A. Snytnikova*, Anton A. Smolentsev, Nataliya G. Kolosova, Anzhella Zh. Fursova, Yuri P. Tsentalovich
*Correspondence: snytnikova_olga@tomo.nsc.ru

Supplementary Information

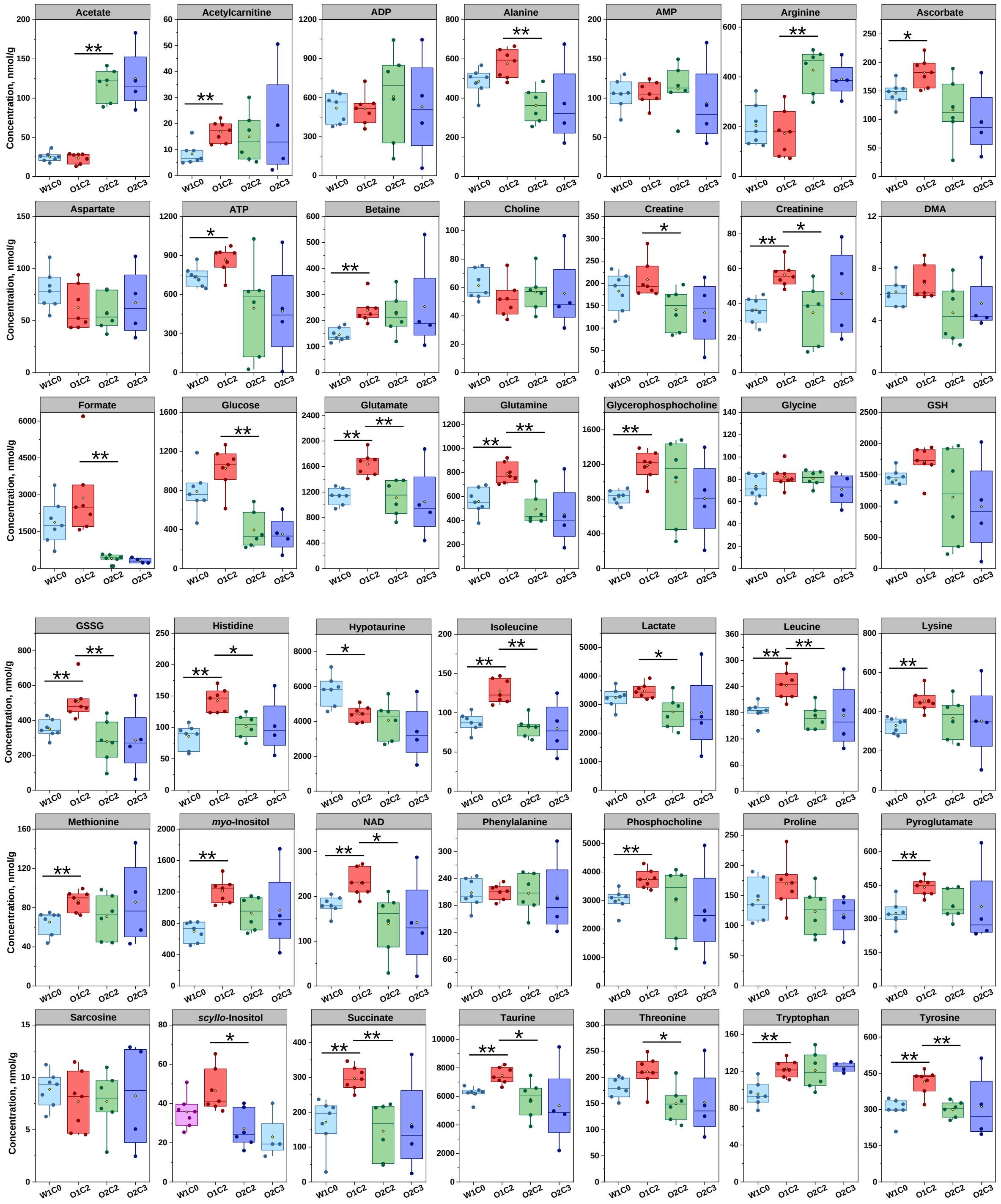

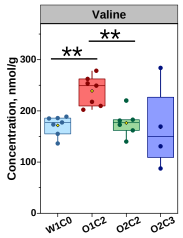

Figure S1. Concentrations of metabolites in the rat lenses. Box plots represent mean value and median ± 1.5 IQR. The metabolite level in lenses is statistically different: ∗ - *p* value < 0.05; ∗∗ - *p* value < 0.01.

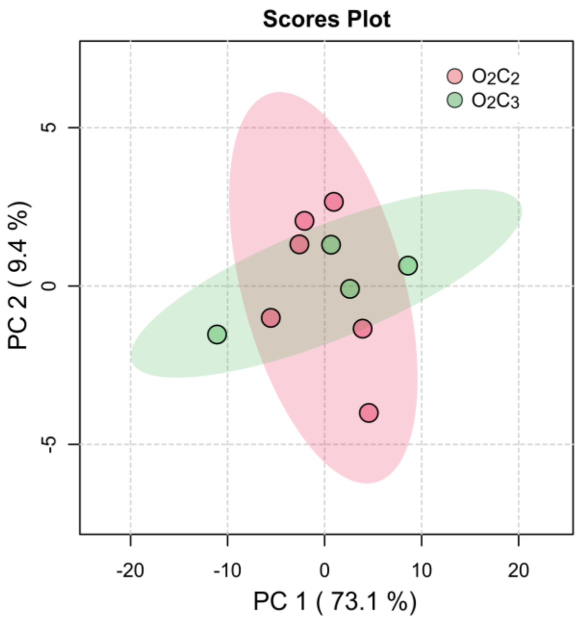

Figure S2. PCA scores plot demonstrating the distribution of metabolomic profiles between groups with different cataract severities (Grade 2 vs. Grade 3). Colored ovals indicate the 95% confidence regions.

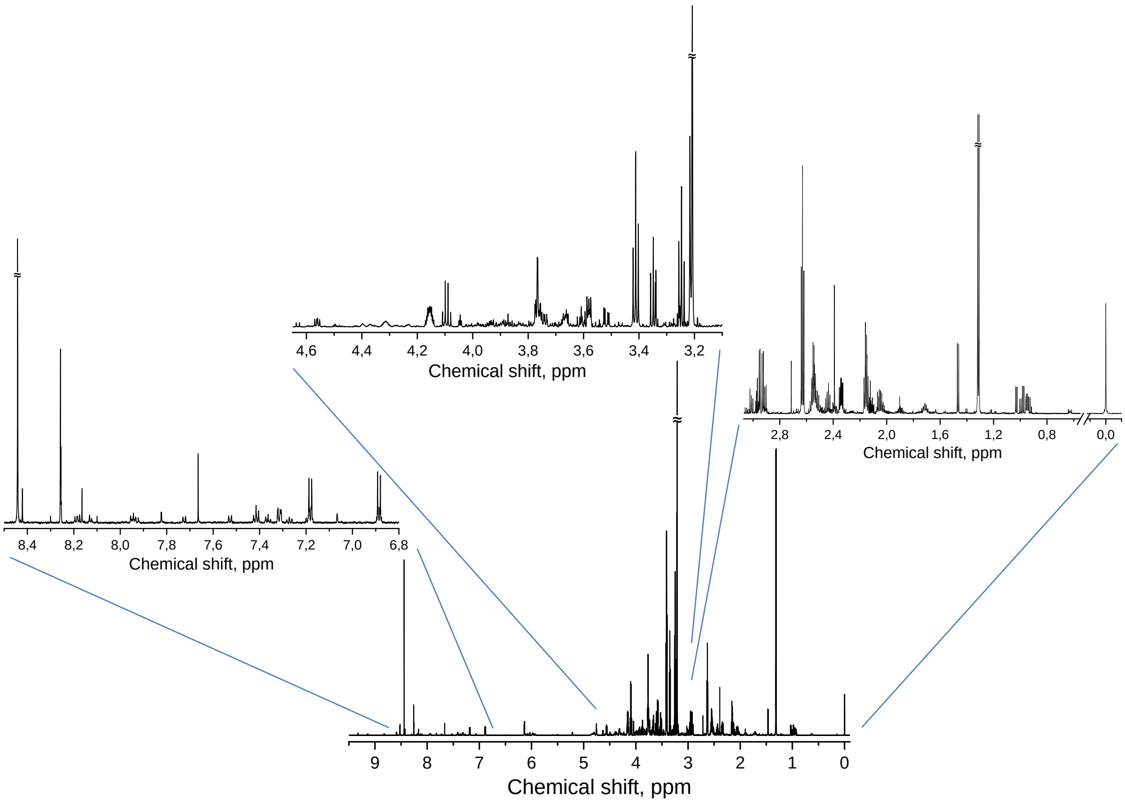

Figure S3. Typical ^{1}H NMR spectrum of a rat lens extract.

Table S1. Concentrations of metabolites in the rat lens. The concentration range from minimum to maximum value, average value with standard deviation are presented, values are given in nmol/g. The following notations were used: **W** (Wistar rats), **O** (OXYS rats); subscripts: **1** (3.6 months), **2** (approx. 4.5 months); **C** (cataract); subscript: grade **0-3**; **N** represents the total number of animals per group.

**Group**	**W_1C_0**		**O_1C_2**		**O_2C_2**		**O_2C_3**	
	N = 7		N = 7		N = 6		N = 4	
	Wistar		OXYS		OXYS		OXYS	
Age, months	3.6		3.6		4.6 ± 0.2		4.2 ± 0.3	
Cataract	Grade 0		Grade 2		Grade 2		Grade 3	
**Metabolite**	**Min - max**	**Mean ± sd**	**Min - max**	**Mean ± sd**	**Min - max**	**Mean ± sd**	**Min - max**	**Mean ± sd**
Acetate ##	17 - 37	25 ± 6	13 - 29	24 ± 7	89 - 141	117 ± 21	85 - 183	130 ± 40
Acetylcarnitine **	5 - 16	8 ± 4	12 - 22	17 ± 4	5 - 30	15 ± 10	2 - 51	20 ± 22
ADP	379 - 649	520 ± 110	360 - 726	514 ± 120	130 - 1042	610 ± 360	58 - 1045	530 ± 410
Alanine ##	363 - 568	490 ± 70	480 - 665	580 ± 70	255 - 485	360 ± 90	171 - 675	370 ± 220
AMP	72 - 130	105 ± 19	81 - 124	106 ± 15	58 - 150	110 ± 30	42 - 171	90 ± 60
Arginine ##	125 - 344	210 ± 80	73 - 321	170 ± 90	298 - 509	430 ± 90	303 - 489	390 ± 80
Ascorbate *	113 - 177	146 ± 20	151 - 222	183 ± 25	29 - 189	120 ± 60	35 - 182	100 ± 60
Aspartate	55 - 111	79 ± 19	43 - 94	63 ± 21	37 - 80	58 ± 18	34 - 112	70 ± 30
ATP *	647 - 871	740 ± 80	671 - 974	870 ± 100	26 - 1027	500 ± 370	7 - 1002	470 ± 410
Betaine **	114 - 185	146 ± 26	189 - 342	240 ± 50	120 - 349	220 ± 80	106 - 531	250 ± 190
Choline	50 - 75	61 ± 10	37 - 76	52 ± 13	39 - 81	57 ± 14	31 - 96	56 ± 28
Creatine #	115 - 232	180 ± 40	177 - 289	210 ± 40	84 - 197	140 ± 50	34 - 214	130 ± 80
Creatinine **, #	25 - 45	36 ± 7	48 - 70	56 ± 7	12 - 56	35 ± 18	19 - 78	46 ± 27
DMA	5 - 8	6.2 ± 1.1	6 - 9	6.9 ± 1.3	2 - 8	4.6 ± 2.3	4 - 9	5.3 ± 2.4
Formate ##	702 - 3389	1900 ± 900	1578 - 6193	2900 ± 1600	100 - 577	410 ± 170	226 - 453	320 ± 110
Glucose ##	466 - 1187	790 ± 220	614 - 1268	1030 ± 210	216 - 686	400 ± 190	136 - 609	350 ± 200
Glutamate **, ##	938 - 1296	1100 ± 100	1410 - 1941	1600 ± 200	726 - 1384	1100 ± 300	440 - 1875	1100 ± 600
Glutamine **, ##	378 - 695	560 ± 110	701 - 921	790 ± 80	395 - 727	500 ± 130	173 - 830	450 ± 280
GPCho **	703 - 926	820 ± 80	889 - 1389	1200 ± 200	312 - 1481	1000 ± 500	211 - 1398	810 ± 490
Glycine	58 - 85	74 ± 11	68 - 101	82 ± 10	70 - 88	81 ± 7	52 - 86	71 ± 15
GSH	1063 - 1692	1400 ± 200	1203 - 1939	1700 ± 300	230 - 1966	1100 ± 800	111 - 2025	990 ± 800
GSSG **, ##	272 - 428	350 ± 50	409 - 724	510 ± 100	95 - 442	280 ± 130	62 - 543	300 ± 200

Histidine **, #	58 - 108	86 ± 19	124 - 170	143 ± 19	74 - 125	100 ± 20	55 - 166	100 ± 50
Hypotaurine *	4574 - 7126	5800 ± 900	3894 - 5097	4500 ± 400	2686 - 5584	4100 ± 1100	1502 - 5718	3400 ± 1700
Isoleucine **, ##	68 - 104	88 ± 11	110 - 147	128 ± 16	65 - 103	82 ± 13	42 - 125	80 ± 40
Lactate #	2640 - 3738	3200 ± 300	3188 - 3926	3500 ± 300	2013 - 3594	2700 ± 600	1191 - 4771	2700 ± 1500
Leucine **, ##	139 - 212	183 ± 22	200 - 293	240 ± 30	142 - 215	170 ± 30	98 - 280	170 ± 80
Lysine **	277 - 375	330 ± 40	382 - 559	460 ± 60	233 - 505	370 ± 110	103 - 609	350 ± 210
Methionine **	44 - 75	65 ± 12	72 - 99	87 ± 10	44 - 98	71 ± 23	43 - 146	90 ± 50
myo – Inositol **	517 – 817	700 ± 130	1028 – 1466	1210 ± 150	673 – 1147	930 ± 220	425 – 1749	970 ± 560
NAD **, #	145 – 205	181 ± 19	189 – 272	230 ± 30	29 – 211	140 ± 70	22 – 287	140 ± 110
Phenylalanine	157 – 245	210 ± 30	184 – 233	210 ± 17	141 – 254	210 ± 50	122 – 323	200 ± 90
Phosphocholine **	2292 – 3503	3000 ± 400	3368 – 4294	3800 ± 300	1312 – 4078	3000 ± 1200	819 – 4931	2700 ± 1700
Proline	104 – 190	140 ± 30	113 – 239	170 ± 40	77 – 178	120 ± 40	73 – 148	120 ± 30
Pyroglutamate **	244 – 422	320 ± 50	384 – 500	440 ± 40	276 – 442	360 ± 70	233 – 639	360 ± 190
Sarcosine	6 – 11	8.9 ± 1.7	5 – 11	7.7 ± 2.8	3 – 11	7.7 ± 2.9	2 – 13	8 ± 5
scyllo – Inositol #	25 – 51	36 ± 8	36 – 65	47 ± 11	16 – 40	27 ± 10	13 – 40	23 ± 12
Succinate **, ##	28 – 237	170 ± 70	249 – 347	300 ± 30	49 – 223	150 ± 80	24 – 366	160 ± 150
Taurine **, #	5235 – 6749	6200 ± 500	6643 – 8230	7500 ± 600	3892 – 7468	5800 ± 1300	2199 – 9467	5300 ± 3000
Threonine #	151 – 203	180 ± 19	153 – 249	210 ± 30	108 – 208	150 ± 40	86 – 252	150 ± 70
Tryptophan **	77 – 117	96 ± 13	111 – 137	123 ± 9	97 – 149	121 ± 20	118 – 130	125 ± 5
Tyrosine **, ##	209 – 347	300 ± 50	320 – 468	420 ± 50	255 – 342	300 ± 30	198 – 513	310 ± 150
Valine **, ##	136 – 189	172 ± 19	202 – 278	240 ± 30	140 – 220	180 ± 30	87 – 284	170 ± 80

* The levels in lenses of Wistar and OXYS rats at the age of 3.6 months are statistically different with p value < 0.05; ** with p value < 0.01.
# The levels in lenses of 3.6- and 4.5-month-old OXYS rats with grade 2 cataracts are statistically different with p value < 0.05; ## with p value < 0.01.
Abbreviation:

AMP – Adenosine monophosphate; ADP – Adenosine diphosphate; ATP – Adenosine triphosphate; DMA – Dimethylamine; GPCho – Glycerophosphocholine;

GSH – Reduced Glutathione; GSSG – Oxidized Glutathione; NAD – Nicotinamide Adenine Dinucleotide

Table S2. ^{1}H NMR spectral data: chemical shifts and signal multiplicities of metabolites identified and quantified in lenses of Wistar and OXYS rats.

**Metabolite**	**Chemical shift (δ, ppm)**	**Metabolite**	**Chemical shift (δ, ppm)**
Acetate	1.902 (s)	Histidine	7.06 (s)
Acetylcarnitine	3.181 (s)	Hypotaurine	2.629 (t)
ADP	8.255 (s)	Isoleucine	0.927 (t)
Alanine	1.466 (d)	Lactate	4.094 (q)
AMP	8.597 (s)	Leucine	0.954 (dd)
Arginine	3.231 (t)	Lysine	3.013 (t)
Ascorbate	4.497 (d)	Methionine	2.123 (s)
Aspartate	2.803 (dd)	*myo* – Inositol	4.045 (t)
ATP	8.257 (s)	NAD	8.421 (s)
Betaine	3.253 (s)	Phenylalanine	7.416 (t)
Choline	3.189 (s)	Phosphocholine	3.209 (s)
Creatine	3.024 (s)	Proline	4.118 (dd)
Creatinine	3.031 (s)	Pyroglutamate	2.39 (m)
DMA	2.698 (s)	Sarcosine	2.725 (s)
Formate	8.442 (s)	*scyllo* – Inositol	3.332 (s)
Glucose	4.631 (d), 5.218 (d)	Succinate	2.391 (s)
Glutamate	2.342 (m)	Taurine	3.412 (t)
Glutamine	2.441 (m)	Threonine	3.57 (d)
Glycerophosphocholine	3.215 (s)	Tryptophan	7.527 (d)
Glycine	3.541 (s)	Tyrosine	6.886 (d)
GSH	4.56 (dd)	Valine	1.031(d)
GSSG	3.298 (dd)		

Table S3. Wilcoxon rank-sum test on correlations of metabolite abundances in the experimental groups.

	W_1C_0 - O_1C_2				O_1C_2 - O_2C_2				O_2C_2 - O_2C_3			
**Metabolites:**	**V**	**p.value**	**-log10(p)**	**FDR**	**V**	**p.value**	**-log10(p)**	**FDR**	**V**	**p.value**	**-log10(p)**	**FDR**
Acetate	26	0.9015	0.0450	0.9015	**0**	**0.0012**	**2.9335**	**0.0095**	13	0.9143	0.0389	1
Acetylcarnitine	**46**	**0.0041**	**2.3894**	**0.0083**	25	0.6282	0.2019	0.7696	12	1	0	1
ADP	21	0.7104	0.1485	0.7735	15	0.4452	0.3514	0.5896	13	0.9143	0.0389	1
Alanine	39	0.0728	1.1376	0.1115	**41**	**0.0023**	**2.6325**	**0.0095**	14	0.7619	0.1181	1
AMP	23	0.9015	0.0450	0.9015	15	0.4452	0.3514	0.5896	16	0.4762	0.3222	1
Arginine	18	0.4557	0.3413	0.5193	**1**	**0.0023**	**2.6325**	**0.0095**	15	0.6095	0.2150	1
Ascorbate	**43**	**0.0175**	**1.7574**	**0.0329**	36	0.0350	1.4564	0.0685	16	0.4762	0.3222	1
Aspartate	14	0.2086	0.6806	0.2840	23	0.8357	0.0780	0.9306	12	1	0	1
ATP	**42**	**0.0262**	**1.5813**	**0.0476**	35	0.0513	1.2900	0.0966	15	0.6095	0.2150	1
Betaine	**49**	**0.0006**	**3.2345**	**0.0026**	24	0.7308	0.1362	0.8526	13	0.9143	0.0389	1
Choline	13	0.1649	0.7827	0.2377	14	0.3660	0.4366	0.5274	14	0.7619	0.1181	1
Creatine	30	0.5350	0.2717	0.5958	**37**	**0.0221**	**1.6547**	**0.0472**	12	1	0	1
Creatinine	**49**	**0.0006**	**3.2345**	**0.0026**	**38**	**0.0140**	**1.8543**	**0.0326**	8	0.4762	0.3222	1
DMA	32	0.3829	0.4170	0.4576	33	0.1014	0.9940	0.1656	9	0.6095	0.2150	1
Formate	35	0.2086	0.6806	0.2840	**42**	**0.0012**	**2.9335**	**0.0095**	18	0.2571	0.5898	1
Glucose	38	0.0973	1.0118	0.1445	**41**	**0.0023**	**2.6325**	**0.0095**	12	1	0	1
Glutamate	**49**	**0.0006**	**3.2345**	**0.0026**	**42**	**0.0012**	**2.9335**	**0.0095**	14	0.7619	0.1181	1
Glutamine	**49**	**0.0006**	**3.2345**	**0.0026**	**40**	**0.0047**	**2.3314**	**0.0152**	15	0.6095	0.2150	1
Glycerophosphocholine	**47**	**0.0023**	**2.6325**	**0.0060**	22	0.9452	0.0245	0.9854	16	0.4762	0.3222	1
Glycine	34	0.2593	0.5862	0.3344	21	1	0	1	17	0.3524	0.4530	1
GSH	41	0.0379	1.4216	0.0599	28	0.3660	0.4366	0.5274	13	0.9143	0.0389	1
GSSG	**48**	**0.0012**	**2.9335**	**0.0036**	**41**	**0.0023**	**2.6325**	**0.0095**	12	1	0	1
Histidine	**49**	**0.0006**	**3.2345**	**0.0026**	**40**	**0.0047**	**2.3314**	**0.0152**	13	0.9143	0.0389	1
Hypotaurine	**4**	**0.0070**	**2.1553**	**0.0137**	24	0.7308	0.1362	0.8526	14	0.7619	0.1181	1
Isoleucine	**49**	**0.0006**	**3.2345**	**0.0026**	**42**	**0.0012**	**2.9335**	**0.0095**	13	0.9143	0.0389	1
Lactate	33	0.3176	0.4981	0.3891	**37**	**0.0221**	**1.6547**	**0.0472**	13	0.9143	0.0389	1

Leucine	**48**	**0.0012**	**2.9335**	**0.0036**	**41**	**0.0023**	**2.6325**	**0.0095**	13	0.9143	0.0389	1
Lysine	**49**	**0.0006**	**3.2345**	**0.0026**	32	0.1375	0.8616	0.2174	13	0.9143	0.0389	1
Methionine	**46**	**0.0041**	**2.3894**	**0.0083**	30	0.2343	0.6303	0.3587	11	0.9143	0.0389	1
Myo-inositol	**49**	**0.0006**	**3.2345**	**0.0026**	34	0.0734	1.1341	0.1285	13	0.9143	0.0389	1
NAD	**46**	**0.0041**	**2.3894**	**0.0083**	**39**	**0.0082**	**2.0884**	**0.0235**	14	0.7619	0.1181	1
Phenylalanine	23	0.9015	0.0450	0.9015	21	1	0	1	14	0.7619	0.1181	1
Phosphocholine	**47**	**0.0023**	**2.6325**	**0.0060**	26	0.5338	0.2726	0.6883	14	0.7619	0.1181	1
Proline	34	0.2593	0.5862	0.3344	34	0.0734	1.1341	0.1285	13	0.9143	0.0389	1
Pyroglutamate	**46**	**0.0041**	**2.3894**	**0.0083**	36	0.0350	1.4564	0.0685	17	0.3524	0.4530	1
Sarcosine	18	0.4557	0.3413	0.5193	20	0.9452	0.0245	0.9854	11	0.9143	0.0389	1
Scyllo-inositol	41	0.0379	1.4216	0.0599	**38**	**0.0140**	**1.8543**	**0.0326**	16	0.4762	0.3222	1
Succinate	**49**	**0.0006**	**3.2345**	**0.0026**	**42**	**0.0012**	**2.9335**	**0.0095**	13	0.9143	0.0389	1
Taurine	**48**	**0.0012**	**2.9335**	**0.0036**	**38**	**0.0140**	**1.8543**	**0.0326**	14	0.7619	0.1181	1
Threonine	41	0.0379	1.4216	0.0599	**38**	**0.0140**	**1.8543**	**0.0326**	13	0.9143	0.0389	1
Tryptophan	**47**	**0.0023**	**2.6325**	**0.0060**	23	0.8357	0.0780	0.9306	11	0.9143	0.0389	1
Tyrosine	**46**	**0.0041**	**2.3894**	**0.0083**	**40**	**0.0047**	**2.3314**	**0.0152**	14	0.7619	0.1181	1
Valine	**49**	**0.0006**	**3.2345**	**0.0026**	**39**	**0.0082**	**2.0884**	**0.0235**	16	0.4762	0.3222	1
**Indices:**	**V**	**p.value**	**-log10(p)**	**FDR**	**V**	**p.value**	**-log10(p)**	**FDR**	**V**	**p.value**	**-log10(p)**	**FDR**
AEC	41	0.0379	1.4216	0.0599	**41**	**0.0023**	**2.6325**	**0.0095**	10	0.7619	0.1181	1
Gln/Glu	23	0.9015	0.0450	0.9015	27	0.4452	0.3514	0.5896	14	0.7619	0.1181	1
Glx	**49**	**0.0006**	**3.2345**	**0.0026**	**42**	**0.0012**	**2.9335**	**0.0095**	14	0.7619	0.1181	1
GSH/GSSG	16	0.3176	0.4981	0.3891	22	0.9452	0.0245	0.9854	13	0.9143	0.0389	1
total choline	**48**	**0.0012**	**2.9335**	**0.0036**	25	0.6282	0.2019	0.7696	14	0.7619	0.1181	1
total GSH	**48**	**0.0012**	**2.9335**	**0.0036**	33	0.1014	0.9940	0.1656	13	0.9143	0.0389	1

p < 0,05

p < 0,01

Table S4. Metabolic indices for rat groups under study: AEC – Adenylate energy charge value; Gln/Glu – index of glutamatergic processing (ratio glutamine to glutamate); Glx – sum of glutamate and glutamine; tCho – sum of glycerophosphocholine, choline and phosphocholine; GSH/GSSG – ratio of reduced glutathione to oxidized glutathione; tGSH – sum of reduced glutathione and oxidized glutathione.

**Group**	$\mathbf{W_1C_0}$	$\mathbf{O_1C_2}$	$\mathbf{O_2C_2}$	$\mathbf{O_2C_3}$
	N = 7	N = 7	N = 6	N = 4
	Wistar	OXYS	OXYS	OXYS
Age (months)	3.6	3.6	4.6 ± 0.2	4.2 ± 0.3
Cataract	Grade 0	Grade 2	Grade 2	Grade 3
**Index**	**Mean ± sd**	**Mean ± sd**	**Mean ± sd**	**Mean ± sd**
AEC	0.734 ± 0.021	0.757 ± 0.018	0.60 ± 0.12	0.59 ± 0.17
Gln/Glu	0.49 ± 0.07	0.49 ± 0.05	0.45 ± 0.08	0.420 ± 0.023
Glx	1700 ± 200	2400 ± 200	1600 ± 400	1500 ± 900
tCho	3900 ± 400	5000 ± 500	4000 ± 2000	4000 ± 200
GSH/GSSG	4.1 ± 0.7	3.5 ± 0.9	4.0 ± 2.4	3.1 ± 1.2
tGSH	1800 ± 200	2000 ± 200	1400 ± 900	1300 ± 1000
